# Anaerobic Exercise Training in the Therapy of Substance Use Disorders: A Systematic Review

**DOI:** 10.3389/fpsyt.2018.00644

**Published:** 2018-12-04

**Authors:** Flora Colledge, Markus Gerber, Uwe Pühse, Sebastian Ludyga

**Affiliations:** Departement für Sport, Bewegung und Gesundheit, Universität Basel, Basel, Switzerland

**Keywords:** exercise, substance use disorder, systematic review, addiction, treatment

## Abstract

**Background:** In the past 3 decades, there has been an increase in the number of studies assessing exercise as a form of treatment for substance use disorders (SUDs). While a variety of substance types and outcomes have been assessed, exercise intensities have never been systematically examined. Consequently, it remains unclear whether particular forms of exercise are better suited to the treatment of these populations. Anaerobic exercise has been shown to have positive effects in populations with psychiatric disorders, but its effectiveness in the treatment of SUDs has to date not been reviewed.

**Methods:** The aim of this systematic review is to identify and evaluate studies which have employed either an acute or chronic anaerobic exercise component as a therapy modality for SUDs. The primary outcomes are abstinence, craving, withdrawal, consumption, quality of life, and the following psychological symptoms and disorders: depression, anxiety, stress, and mood. A secondary objective is to assess whether the type of training described in the study protocol can be reliably categorized as anaerobic training.

**Results:** Twenty-six studies are included in this review. Twelve studies addressed nicotine dependence, one addressed alcohol dependence, and 13 addressed dependence on various illicit drugs. Thirteen studies reported the intensity at which participants actually exercised, but only one employed a test to determine whether training was carried out above the anaerobic threshold (AT). The risk of bias in the included studies was generally high. Results of the studies were mixed, with the most positive effects being found for abstinence in nicotine dependence.

**Conclusion:** The evidence for the effects of anaerobic exercise in SUDs is weak, although a tendency toward positive effects on abstinence in nicotine dependent individuals was observable. The majority of studies do not report data on exercise intensity, making a categorization of anaerobic exercise impossible in all but one case. This means that the effects of this form of exercise cannot be determined, and therefore not evaluated or compared with other forms. In order to improve the quality of evidence for exercise in SUD treatment, clearly defined and objectively assessed evaluations of anaerobic and anaerobic exercise are necessary.

## Introduction

Substance use disorders (SUDs) are severe and persistent psychiatric conditions, which adversely affect the health and social functioning of sufferers, and are a major burden to their families and wider society (1). Globally, 11.5% of deaths can be attributed to smoking (2), and 3.8% to alcohol consumption (3), while illicit drugs account for approximately 0.4% (4). Regarding morbidity, high levels of alcohol consumption are associated with increased risks of developing various cancers, psychiatric disorders, cardiovascular disorders, and sustaining severe injury (5). Smoking has been found to contribute to the development of cancers, cardiovascular and pulmonary diseases, and complications with pregnancy and birth (6). Illicit drug use can lead to non-fatal overdose, HIV and hepatitis infection, and various psychiatric disorders (7). It has been suggested that 91 million family members of sufferers are affected by SUDs (8).

A number of treatment modalities are employed for SUDs. Outpatient psychotherapy, with or without pharmacotherapy, may be employed for almost all SUDS; for alcohol and illicit drugs, inpatient treatment, again involving combinations of psycho- and pharmacotherapy, may be employed (9). The aim of treatment is the management of withdrawal and detoxification (for substances such as alcohol and opioids), and a subsequent sustained reduction or complete cessation (abstinence) of consumption (10). However, relapse following treatment for SUDS is common, with rates of 70–85% reported for smoking (11), alcohol (12), and illicit drugs (13). Consequently, there is a need to identify forms of adjunct therapy which may improve the effectiveness of standard treatment (14).

Exercise is recognized as a key component of health in humans (15, 16), and is increasingly being implemented as a form of adjunct therapy in the most burdensome health complaints (17–19). Evidence for the positive effects of exercise in psychiatric disorders is also increasing (20, 21).

To date, numerous studies have assessed the effects of both acute exercise bouts and long-term exercise programmes as an adjunct therapy in SUDs. The majority of these have focussed on nicotine dependence, with some positive effects found for craving (22), duration of abstinence (23), and withdrawal symptoms (24). However, results of a meta-analysis of randomized controlled trials found no effects for most forms of exercise on smoking cessation, and only low quality evidence for a combination of yoga and cognitive behavioral therapy (25). For alcohol, the results of studies have been mixed, with some reporting a reduction in consumption (26, 27), and others finding no such effects (28) or comorbid psychiatric complaints such as depression (29). A recent meta-analysis concluded that exercise appears to impact positively on depressive symptoms and physical fitness, but does not lead to reductions in consumption (30). The evidence for exercise in the therapy of illicit SUDs is weak, as many studies must contend with small sample sizes and irregular participation (31). At the most, results tend to be promising, with indications that exercise may increase rates of abstinence, reduce psychological complaints, and reduce withdrawal symptoms (32).

In summary, while some positive effects have been found, research into exercise in the therapy of SUDs is still at an early stage. Not only do many studies in the field fall below the strictest criteria of scientific rigor, thorough explorations of mechanisms, and modalities have also yet to be carried out. Specifically, it is important to address the question of whether different forms or intensities of exercise have differing impacts on substance use related outcomes. To date, no review or meta-analysis has sought to differentiate the effects of exercise intensities in populations with SUDs. Furthermore, numerous reviews in this field look only at specific SUDs, and many do not include both chronic and acute outcomes.

A number of theories have suggested that differences in exercise intensity may alter the effects of exercise on psychological health. For instance, Dietrich's transient hypo-frontality theory suggests that following exercise at levels above the individual anaerobic threshold (IAT), there is a downregulation of activity in the pre-frontal cortex due to increasing task demands, but finite resources (33). As neuroimaging studies of depression and anxiety disorders have shown evidence of hyperactivity in this region of the brain, it is hypothesized that exercise above the IAT may lead to a reduction in symptoms via a normalization of the pre-frontal cortex activity (34). Anaerobic sprint training, when compared to steady state running, has also been found to elicit stronger increases in BDNF and dopamine levels (35). Certain drugs of abuse affect the structural plasticity of the brain, and it is hypothesized that BDNF may mediate the structural changes caused by drug exposure (36); consequently, based on findings from pre-clinical studies, it has been hypothesized that exercise-induced increases in BDNF may serve as a substitute reward for drug intake by increasing and adapting dopaminergic activity (37). Finally, vigorous exercise has been shown to influence neurotransmitters, including dopamine (38), the activity of which is involved in the establishment and maintenance of SUDs (39).

In the field of psychological disorders, some studies have found positive effects of exercise which could be classed as anaerobic. Doyne et al. found that both running and weightlifting significantly reduced symptoms of depression in comparison to wait-list controls (40). Martinsen et al. found no differences between individuals with depression who completed aerobic training compared to those who completed strength training, and both groups significantly reduced symptoms of depression (41). Interestingly, as emphasized by Brosse et al., this study may indicate that the effects of exercise on depression are not solely dependent on aerobic conditioning, as only the aerobic training group improved their maximum oxygen uptake (42). Finally, Singh et al. found that high intensity resistance training was effective in reducing symptoms of depression in older adults (43), even when compared to low intensity resistance training (44). It is therefore important that the extent to which anaerobic training has been employed in the treatment of SUDS is explored, in order to understand its effects. No systematic assessment of the implementation of this intensity of exercise has, to our knowledge, been carried out so far; consequently, the effects in SUD populations remain unclear.

The aim of this systematic review is to document the implementation of anaerobic exercise in the treatment of SUDs. The term anaerobic is subject to some ambiguity (45). We therefore provide definitions of this and other key terms below.

## Definitions of Aerobic and Anaerobic Exercise

Aerobic training or exercise is defined as movement of large muscle groups performed at an intensity which keeps the athlete below the anaerobic threshold (AT) (46). Anaerobic training, in turn, is exercise carried out above the AT, and therefore powered by metabolic pathways not solely dependent on oxygen (47). This threshold has also been termed the ventilatory threshold or lactate threshold (48). The AT is defined as the intensity at which lactate accumulates in the blood more quickly than it can be cleared, leading to the onset of impairments in exercise performance (49). Training above the AT therefore cannot be continued indefinitely, but will lead to exhaustion and inability to continue exercising. Originally, it was supposed that a lactic acid concentration of 4 mmol/l corresponded with this threshold (50). However, it was soon determined that this level varies individually, and consequently that it is more suitable to speak of the IAT when assessing exercise intensity (51). The IAT tends to be lower among untrained individuals (52).

Frequently, heart rate during exercise will be used as a further defining criterion between aerobic and anaerobic training, as this measure can be far more simply and quickly assessed by the individual than plasma lactate concentration (53). Unfortunately, heart rate at the AT is also individual, so definitions based on this parameter are also problematic (54). Consequently, exercise defined as vigorous or intense may bring an untrained individual to or beyond their IAT, despite the heart rate achieved during training falling below the IAT of a well-trained individual.

As a result, it is not accurate to define a particular type of exercise, such as running, as aerobic or anaerobic, because the threshold at which lactate accumulates in the blood is dependent on the individual, and hence can only be determined with physiological testing (55). However, in daily usage and even in the scientific literature it has become commonplace to use both terms to refer to particular exercise types. Aerobic training is typically used to designate exercise of low to moderately high intensity, often carried out for at least 20 min, and is often attributed to walking, light jogging and cycling (56). Anaerobic training is used to refer to high intensity exercise, typically in short and repeated bouts, such as sprinting (57), interval training (58), and weight lifting (59). They key problem with such designations is that, particularly in untrained populations, an activity such as running may frequently result in the surpassing of the IAT, meaning that the training was not solely “aerobic” at all.

In this paper, the term “anaerobic” refers to exercise which brings an individual to or beyond their IAT, as measured by concentration of lactic acid in the blood. However, given the issues of taxonomy noted above, we suspected that a number of studies which involve exercise intensities at or above the AT might not use the term “anaerobic.” Consequently, in order to avoid overlooking studies which may involve training above the IAT, but which do not provide the physiological data to confirm this, we used a variety of search terms and inclusion criteria. These are intended to ensure that all studies in which participants may have exercised at or above the IAT are included in this review.

## Objectives

The aim of this review is to identify and evaluate studies which have employed either an acute or chronic anaerobic exercise component as a therapy modality for SUDs, in order to summarize what is currently known about the effects of this exercise intensity. The primary outcomes are abstinence, craving, withdrawal, consumption, quality of life, and the following psychological symptoms and disorders: depression, anxiety, stress, and mood. A secondary objective is to assess whether the type of training described in the study protocol can be reliably categorized as anaerobic training.

## Methods

The review was planned and carried out in line with the guidelines for the Preferred Reporting Items for systematic Reviews and Meta-Analyses (PRISMA) (60), and registered at the international prospective register of systematic reviews PROSPERO (http://www.crd.york.ac.uk/PROSPERO/display_record.php?ID=CRD42017082658).

## Inclusion and Exclusion Criteria

Included in the review are all studies which, as a form of treatment for SUDs, involved acute or chronic exercise of the following type: (1) reported by authors as being at or above the specifically determined IAT; (2) at or above a heart rate which corresponds to 75% of maximum, (3) at or above 70% of heart rate reserve, (4) at a score of 14 or above on the Borg scale, (5) described by the authors as vigorous, intense, or anaerobic, or (6) involving activities which may incorporate bouts above the IAT, where the authors do not explicitly state that this was not the case, by reporting physiological data (for example, heart rate below 75% of maximum). These activities are defined as any type of sport, exercise or structured physical activity excluding yoga, Pilates, stretching, walking, medical rehabilitation, Qi Gong, or Tai Chi. Exercise reported by the authors to be “aerobic” or “moderate” was not an automatic exclusion criterion, as many forms of exercise referred to as “aerobic” in common parlance may bring untrained individuals to or above the IAT for a period of time.

Types of substance included are cigarettes, alcohol, and all illicit and prescription drugs. Authors were contacted to provide any information pertaining to the above criteria, outcomes, and potential sources of bias. If authors did not respond once, they were contacted again 2 weeks later.

The primary endpoint of this review is the effect of anaerobic exercise on abstinence (or the absence of relapse), craving, consumption, quality of life, and the following psychological symptoms and disorders: depression, anxiety, stress, and mood. Consequently, studies which assessed only other outcomes, such as strength and fitness, were excluded. Studies not in English, German, or French were also excluded.

## Search Strategy

Three researchers were involved in the search and inclusion strategy. Two authors (FC and SL) searched the databases MEDLINE, Web of Science, Google Scholar, SPORTDiscus and PsycINFO from database inception to the 15th of November 2017. The following terms were used in the search: “anaerobic” or “exercise” or “intense exercise” or “vigorous exercise” or “anaerobic training” or “strength training” or “resistance training” or “physical activity” and “substance abuse” or “substance” or “substance use” or “addiction” or “drug abuse” or “drug” or “addiction” or “dependence” or “alcohol” or “illicit” or “smoking” or “nicotine.” Manual searches of the reference lists of articles were also carried out. Following removal of duplicates, two authors (FC and SL) read the titles and abstracts to assess eligibility based on the exclusion criteria. In some cases, the full text was read in order to determine if the criteria were met. The final list of studies to include was the result of the two independently developed lists of the authors, agreed upon by discussion. The third author (MG) resolved differences of opinion.

## Data Extraction

Two authors (FC and SL) independently extracted the following data: substance type, substance consumption (amount per day, amount per year, and years of consumption), primary diagnosis, sample size and characteristics, duration, quality, exercise characteristics, reported goal exercise intensity, reported achieved exercise intensity, control condition, and the following study outcomes: abstinence (absence of relapse), craving, withdrawal, consumption, quality of life, and psychological symptoms and disorders (stress, anxiety, depressions, and mood).

## Risk of Bias

One author (FC) assessed studies for risk of bias in the categories defined in the Cochrane risk of bias tool (61), excluding the items on blinding participants and investigators, which were deemed inappropriate for trials of exercise. Each study received a score of low, high or unclear risk of bias in each category.

## Results

**Figure 1:**
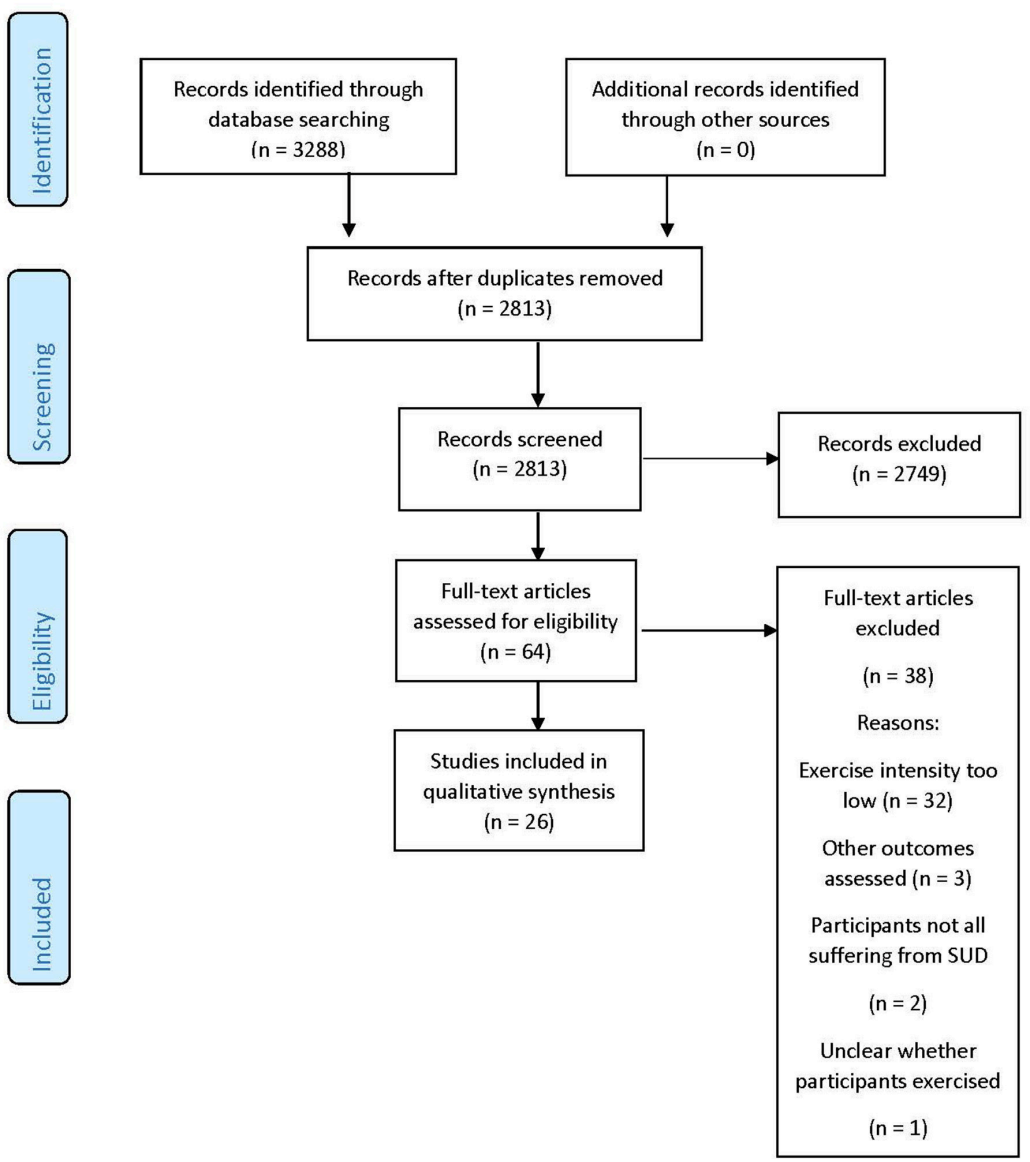
PRISMA flow diagram.

Details of the literature search are provided in Figure 1.

### Study Selection and Characteristics

Twenty-six studies are included in this review. A summary of the study characteristics can be found in Table 1. Sample sizes ranged from 18 (69) to 330 (71), with a mean sample size of 97. Mean age was 34.3 years. Twelve studies addressed nicotine dependence (62–73), one addressed alcohol dependence (74), and 13 addressed dependence on various illicit drugs (75–87). Four studies involved acute exercise bouts (67, 69, 84, 85), and the rest involved long-term interventions, lasting between 7 weeks (66) and 15 months (78). Total exercise sessions (excluding warmup and cooldown) lasted from 10 min (67) to 120 min (77). Thirteen studies reported the intensity at which participants actually exercised; four studies used the Borg scale (67, 68, 82, 85), one study included self-reported time spent in vigorous exercise (66), two studies stated that goal intensity had been reached without further detail (72, 79), and seven studies provided physiological measures (63–65, 69, 78, 84) [of which one also reported Borg values (67)]. Of these, four studies reported mean heart rate (63–65, 84), two reported mean heart rate reserve (67, 69), and one reported time spent above the predetermined IAT (78). Five studies carried out a within-subjects assessment only (65, 69, 77, 78, 88), and the rest studies included control groups; three of these included two control groups (62, 67, 68), one included three (84), and one included a control group but also carried out a within-subjects analysis (85). In the control conditions, two carried out aerobic exercise (67, 84), three received treatment as usual (68, 74, 79), and the rest received a non-exercise intervention.

**Table 1:** Studies on the effect of exercise on nicotine dependence.

**References**	**Duration**	**Participants in exercise arm**	**Control condition^a^**	**Goal exercise**	**Achieved intensity reported**	**Outcomes^b^**	**Abstinent at study start**
Russell et al. (62)	9 sessions	42 females M age: 28 (*SD*: 7) years M cigarettes per day: 23 (*SD*: 7) M years smoking: 10 (*SD*: 4)	BS: 2 groups: habit change, attention control	1 supervised h and 2 recommended 20–30 sessions per week including walk/jog at 70–80% of HR max	No	3, 6, 18 month abstinence ↑ Anxiety - ↑	No
Marcus et al. (63)	15 weeks	10 females M age: 40 (*SD*: 9) years M cigarettes per day: 27 (*SD*: 11)	BS: 4 weeks of twice weekly behavioral modification training	3 sessions per week of indoor cycling at 70–85% HR max	Mean 80% of HR max	7 day abstinence ↑	No
Marcus et al. (64)	12 weeks	134 women M age: 40.7 (*SD*: 9.1) years M cigarettes per day: 22.9 (*SD*: 9.7) M years smoking: 22.6 (*SD*: 8.8)	BS: quit and weight loss promotion	3 sessions per week of 30–40 min activity at resting heart rate plus 60–85% of heart rate reserve	Mean 83% of HR max	8, 20, and 60 week abstinence ↑	No
Bock et al. (65)	See (64)	See (64)	WS	See (64)	See (64)	Acute post-training negative affect, craving, withdrawal symptoms ↓	No
Ussher et al. (66)	7 weeks	154 adults M age: 41.5 (*SD* 11.1) years M cigarettes per day: 21.6 (*SD*: 8.8)	BS: equal staff contact time	Exercise counseling promoting vigorous physical activity	0.1–0.3 h per week vigorous physical activity self-reported	Anxiety and stress 6 week abstinence -	No
Everson et al. (67)	Acute bout	15 adults M age: 21.53 (*SD*: 2.39) years M cigarettes per day: 13.93 (*SD*: 3.96)	BS: 2 groups: moderate aerobic cycling, passive waiting	10 min of cycle ergometry at 60–84% of heart rate reserve or 14–16 Borg scale	Mean 67.9% heart rate reserve, 14.8 (Borg)	Craving compared to passive control only ↓ Depression -	Yes
Kinnunen et al. (68)	19 weeks	75 women M age: 38.3 (*SD* 9.9) years M cigarettes per day: 18.5 (*SD* 8.0)	BS: 2 groups: equal staff contact time, standard care	1–2 sessions per week (up to 3 recommended) of walking/jogging at 60–80% HR max	Self-reported RPE (Borg) used but not reported	Abstinence, withdrawal, depression -	No
Scerbo et al. (69)	Acute bout	18 adults (8 females) M age: 26.0 (*SD*: 4.2) years M cigarettes per day: 13.9 (*SD*: 3.6)	WS: 2 groups: passive waiting, walking	15 min of treadmill running at 80–85% HR reserve	Mean 81.5% HR reserve	Craving compared to passive control only ↓	Yes
Ciccolo et al. (70)	12 weeks	13 females For total sample: M age: 36.5 (*SD*: 12.0) years M years smoking: 19.1 (*SD*: 12.0) M cigarettes per day: 18.0 (*SD*: 10.1)	BS: short health film	2 sessions per week of 60 min, 1–2 sets of 10 repetitions of full body resistance programme, weight adjusted to 75% max strength.	No	7-day point prevalence abstinence at 3 and 6 months ↑	No
Whiteley et al. (71)	12 weeks	166 females M age: 44.1 (*SD*: 9.9) M cigarettes per day: 17.4 (8.0)	BS: equal staff contact time	(From week 5)3 sessions per week recommended of at least 40 min at 77–85% HR max	No	7-day point prevalence abstinence, 3, 6, and 12 month abstinence -	No
Smits et al. (72)	15 weeks	72 adults (36 female) M age: 43.1 (*SD*: 11.3) years M cigarettes per day: 16.9 (*SD*: 7.8)	BS: health education	3 sessions per week of 35 min at 77–85% HR max	94% of participants achieved goal intensity	7-day point prevalence abstinence, 4 and 6 month abstinence ↑ only for participants with high anxiety sensitivity	No
Patten et al. (73)	12 weeks	15 females M age: 37.0 (*SD*: 10.0) years	BS: health education	3 sessions per week of 20–30 min of exercise on cardiovascular equipment with progressively longer periods at vigorous intensity of 5–8 RPE	No	Abstinence at study end ↑ Abstinence at 6 months -	No

a *BS, between subjects; WS, within subjects*;

b *outcomes and, unless otherwise stated, direction of significant effect:↑ = increase, ↑ = decrease, - =, no difference*.

### Excluded Studies

A number of studies were excluded after review of the full text (several more having been excluded following reading of the abstract). Three studies involved an exercise bout or intervention for individuals with SUDS which fit our inclusion criteria for vigorous or anaerobic exercise (76, 89–91); however, primary outcomes other than those which are the focus of this review were assessed. In four studies, anaerobic or vigorous exercise was the goal intensity, but it was explicitly reported that participants did not achieve this intensity (92–94). In the case of Williams et al. although it was reported that 81% of subjects achieved an exercise intensity of 64–76% of HR max, a maximum value surpassing our minimum inclusion criteria by 1%, the mean achieved intensity was reported to be 68% (88). In two studies, all participants were not diagnosed with an SUD, but were reported to be “heavy drinkers” (95), or represented a mix of smokers and non-smokers (96). In one study, participants in the exercise programme did not in fact all participate in the sport (softball), as some took on roles such as coach or videographer (97). Finally, in one study, neither the exercise intensity, nor the type, was reported (98).

## Synthesized Findings

### Nicotine Dependence

Studies addressing nicotine dependence are presented in Table 1.

Nine studies, all focusing on chronic exercise, reported abstinence as an outcome. Of these, four studies reported an increase at all time points compared to the control condition (62–64, 70). One study reported an increase at study end, but not at the 6-month follow-up (73). One study reported an improvement in only those participants characterized *post-hoc* as having high anxiety sensitivity (72). Three studies reported no improvement in this domain (66, 68, 71).

Three studies reported craving as an outcome. In the first study, during and 5 min after an acute bout of cycling, craving was reduced in comparison to a passive control condition (67). In the second study, this pattern of results following an acute bout was repeated, but craving remained reduced up to 30 min post-exercise (69). In the third study, craving was reduced following a chronic exercise programme, but there was no control condition (65).

Two studies reported withdrawal symptoms as an outcome. One found reductions immediately following exercise, but not from baseline to study end (65); the other reported no improvements (68).

Two studies reported anxiety as an outcome, with one reporting a reduction (66), and the other no change (62), compared to controls.

One study reported stress as an outcome, and a reduction in comparison to the control condition was reported (66).

Two studies reported depression as an outcome; neither the acute (67) nor the chronic intervention (68) led to improvement compared to the control condition.

One reported mood. Bock et al. found acute reductions in negative affect immediately following exercise (65).

### Alcohol Dependence

Studies addressing alcohol dependence are presented in Table 2.

**Table 2:** Studies on the effect of exercise on alcohol dependence.

**References**	**Diagnosis**	**Duration**	**Participants in exercise arm**	**Control condition^a^**	**Goal exercise**	**Achieved intensity reported**	**Outcomes^b^**	**Abstinent at study start**
Roessler et al. (74)	Alcohol dependence (ICD – 10)	6 months	Group: 62 adults (24 females) M age: 44.8 (*SD*: 11.2) years Alone: 60 adults (15 females) M age: 43.8 (*SD*: 11.1)	BS: treatment as usual	Group: 1–2 per week sessions of 25–45 min walking/running in group with intervals at 80–90% HR max Alone: as above without group	No	Excessive alcohol consumption -	No

a *BS, between subjects, WS, within subjects*;

b *outcomes and, unless otherwise stated, direction of significant effect:↑ = increase, ↓ = decrease, − = no difference*.

The single study involving primarily alcohol dependent participants reported consumption (excessive drinking) as an outcome. No difference was found in comparison to the control group (74).

### Drug Dependence

Studies addressing drug dependence are presented in Table 3.

**Table 3:** Studies on the effect of exercise on drug dependence.

**First author, year**	**Substance**	**Diagnosis**	**Duration**	**Participants in exercise arm**	**Control condition^a^**	**Goal exercise**	**Achieved intensity reported**	**Outcomes^b^**	**Abstinent at study start**
Williams et al. (75)	Various drugs, alcohol	?	12 weeks	20 adults (7 female) M age: 30.0 years	WS	2 sessions per week of 45 min strength training	No	Anxiety and depression - →	?
Williams et al. (76)	Opioids	Opioid dependence	4 months	9 men M age: 37.0 (*SD*: 3.0) years More than 5 years opioid dependence	BS: *post-hoc* classification of exercise completers and non-completers	3 sessions per week of 70 min including cycle ergometer at up to 80% HR max and strength training	No	Quality of life -	60 mg methadone
Roessler (77)	Various drugs	Substance dependence diagnosed by clinic	2–6 months	38 adults (15 female) M age: 35.00 years	WS	3 sessions per week of 2 h including cycle ergometer and strength training	No	No inferential statistical assessment (abstinence)	No
Mamen (78)	Various drugs	Substance abuse/dependence (DSM–IV)	2–15 months	33 adults (7 female) M age: 31.2 (*SD*: 9.9) years	WS	“Almost daily” session with trained supervisor including jogging, cycling, team sports	74% of training time below lactate threshold	Depression and anxietyConsumption- ↓	?
Flemmen et al. (79)	Various drugs	Substance dependence (ICD−10: F10-F19)	8 weeks	9 (1 female) M age: 33 (*SD*: 11) years M years drug abuse: 17.0 (*SD* = 8.0)	BS: treatment as usual (including activity reported at approx. 70% HR max)	3 sessions per week of 4 times 4 min walking/running at 90–95% HR max	Target intensity reportedly reached	Anxiety and depression -	?
Rawson et al. (80)	Meth-amphetamine	Methamphetamine dependence (DSM–IV)	8 weeks	69 adults (21 females) M age: 31.9 (*SD* = 7.4) years M Methamphetamine use days: 15.9 (*SD* = 9.1)	BS: health education	3 sessions per week of 60 min including 30 min treadmill at 60–80% HR max and 15 min strength training	No	Depression, anxiety ↓	Yes
Rawson et al. (81)	Meth-amphetamine	See (80)	See (80)	See (80)	See (80)	See (80)	No	Consumption in low severity users at 1, 3, and 6 months ↓	Yes
Gimenez-Meseguer et al. (82)	Various drugs	Substance dependence (DSM-IV)	12 weeks	18 adults (4 female) M age: 37.2 (*SD* = 6.8) years	BS: free time	3 sessions per week of 60–90 min	M perceived intensity 4.4 (modified 10 point Borg)	Dimensions of quality of life ↑	Yes
Unhjem et al. (83)	Various drugs	Substance use disorder (ICD-10: F10–F19)	8 weeks	9 adults (3 females) M age: 33.0 (*SD* = 9.0) years M years drug use: 13.0 (*SD* = 10.0)	BS: activities	3 sessions per week of strength training at 85–90% of 1 rep max for 4 sets of 4–5 reps	No	Anxiety (within group only) ↓ Depression -	No
Wang et al. (84)	Meth-amphetamine	Substance dependence (DSM–IV)	Acute bout	23 adults (4 female) M age: 34.0 (*SD* = 5.9) years M methamphetamine per use: 0.56 (*SD* = 0.4) grams	BS: 3 groups: passive control, exercise at 40–50% of HR max, exercise at 65–75% HR max	20 min at 85–95% HR max on cycle ergometer	M HR achieved: 167.4 (*SD*: 6.9) BPM	Craving during exercise compared to light intensity and control, and up to 50 min after exercise compared to control ↓	Yes
Grandjean da Costa et al. (85)	Various drugs	Substance dependence (DSM–V)	Acute bout	14 adults M age: 33.0 years (*SD* = xx.x)	WS and BS: healthy controls	Maximal exercise test on cycle ergometer until 18–20 on Borg scale or exhaustion	Borg 19+	Craving *post-test* ↓	Yes
Trivedi et al. (86)	Various drugs	Substance dependence (DSM–IV)	12 weeks	152 adults (61 females) M age: 38.5 (*SD* = 10.0) years	BS: health education	3 sessions per week treadmill running at 70–80% of HR max from week 3	No	Abstinence -	Yes
Colledge et al. (87)	Opioids	Opioid dependence diagnosed by clinic	12 weeks	13 adults (4 females) M age: 42.7 (*SD* = 6.5) years M diacetylmorphine per day: 377 (*SD* = 164) mg	BS: equal staff contact time	2 sessions per week including boxing, badminton, climbing, strength training	No	Depression, stress, consumption -	No

a *BS, between subjects; WS, within subjects*;

b *outcomes and, unless otherwise stated, direction of significant effect:↑ = increase, ↑ = decrease, - =, no difference*.

Two studies reported abstinence as an outcome. Of these, one found no difference between exercisers and controls upon study completion (86). The other carried out no inferential statistical assessment, but reported that 5 of 20 exercisers reported abstinence at 1 year follow-up (77).

Two studies, both involving acute exercise bouts, reported craving as an outcome. Wang et al. found that, compared to a bout of light exercise or non-exercising controls, craving was reduced during and up to 50 min post-exercise, though there was no difference compared to a moderate intensity bout (84). Grandjean da Costa et al. reported a reduction immediately following the exercise bout (85).

Three studies reported consumption as an outcome. Two found no difference, in one case within subjects (78), in the other compared to controls (87). The second found a reduction in low severity users (based on pre-treatment use) only at 1, 3, and 6 months follow-up (81).

Two studies reported dimensions of quality of life as outcomes. One found no difference compared to controls (76), while the other reported improvements compared to controls (82).

Six studies reported depression as an outcome. In three studies, no difference compared to the control group was found (79, 83, 87); in one, no change within subjects was observed (75); and in two, a reduction in comparison to the control group was found (78, 80).

Five studies reported anxiety as an outcome. One study found no difference between exercise programme completers and dropouts (75); one found no difference to the control group (79); two found a reduction in comparison to the control condition (78, 80), and one reported a reduction within subjects (83).

One study reported stress as an outcome. Colledge et al. found no differences compared to non-exercising controls (87).

### Risk of Bias

**Figure 2:**
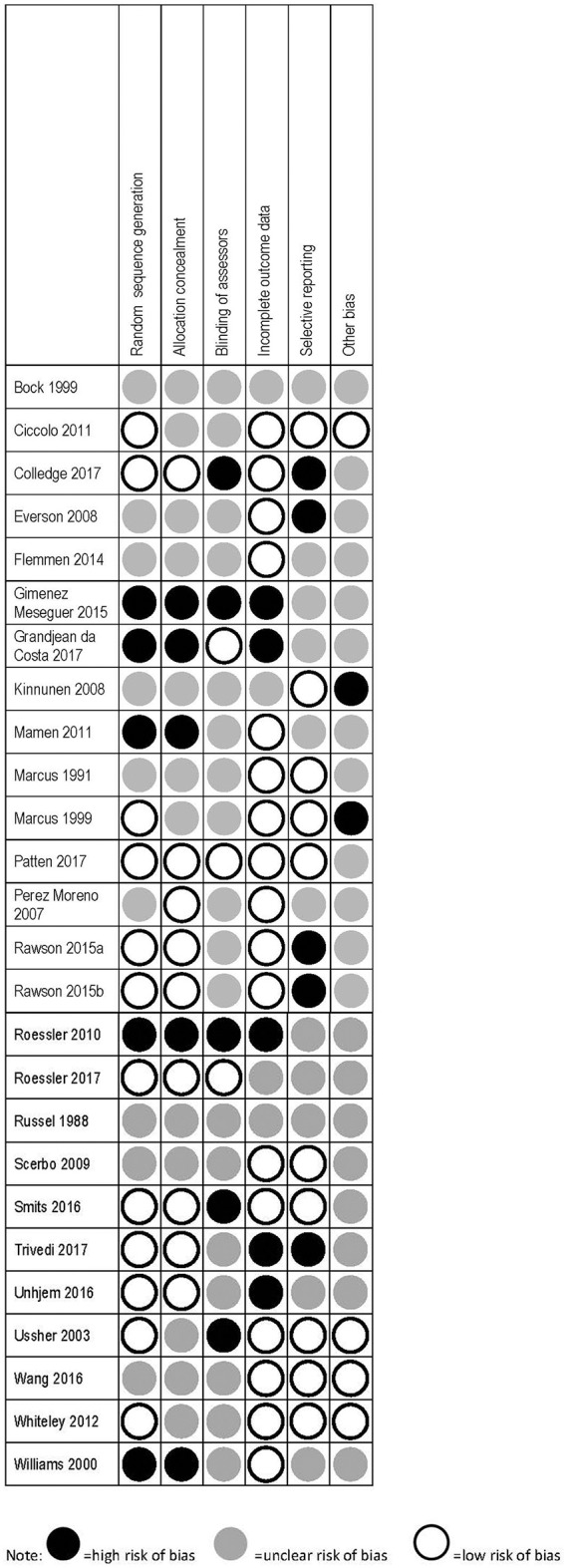
Risk of bias assessment based on modified cochrane risk of bias tool.

Details of the risk of bias assessment are provided in Figure 2.

No study achieved a low risk of bias across all categories. Eleven studies had low risk in three or more of the six possible categories (64, 66, 70–74, 80, 81, 84, 87), 11 studies had low risk in one to two categories (63, 67–69, 76, 78, 79, 83, 85, 86, 88), and four had no low risk categories (62, 65, 77, 82). The scoring outcomes are shown in detail in Figure 2. The studies included in this review incorporate heterogeneous designs, patient groups, and outcomes. Participants in the various studies were in some cases receiving pharmacological treatment (e.g., nicotine patches, methadone), or were residents at an inpatient treatment facility. In all studies except those focusing on nicotine dependence, exercise is therefore employed as an adjunct form of treatment. It must also be noted that research into exercise is likely to involve a certain selection bias, as individuals unwilling or unable to participate will not be represented.

## Discussion

To our knowledge, this is the first review to focus exclusively on anaerobic exercise as a treatment modality in SUDs, and the first to address the difference between aerobic and anaerobic exercise types at all. This distinction is important, because aerobic and anaerobic training involve different physiological processes, which may differentially impact on outcomes relevant in SUDs. If these two exercise types are not assessed separately, the mechanisms through which exercise may improve these outcomes remains unclear.

Only a single study addressing alcohol dependence fit our criteria, and while 13 were found for illicit drugs, it must be emphasized that only a small number addressed the same specific drugs, such as methamphetamine or heroin. Consequently, no conclusions concerning effect trends can be drawn; rather, the key finding for these substances is that studies which clearly define exercise intensities, and test protocols above the AT, are required.

## Summary of Main Findings Regarding Primary Outcomes

The findings of this review suggest that anaerobic or vigorous exercise may have a positive effect on a number of outcomes in populations with a SUD. The term vigorous must be employed here, as in all but one study, it cannot be stated with certainty that participants met or exceeded their IAT at any point. However, the quality of the evidence is generally weak, and in 9 of the 26 included studies, no improvement in any outcome included in this review was detected. Conclusions, and indeed speculations about hypothesized mechanisms influenced by anaerobic training, such as activity of the pre-frontal cortex (33), or BDNF secretion (35), are not possible. The following discussion of the results must be considered in light of these facts.

Of the 12 studies in the review which address nicotine dependence, the most commonly reported outcome was abstinence, with six of the nine studies assessing this parameter reporting at least some positive effects. This is the most positive finding of this review. While no conclusions about this effect can be drawn on the basis of this data, there are a number of plausible speculative explanations. Individuals who are prepared to begin, and comply with, an exercise programme, may also have the capacity to commit to challenging changes in other area, such as quitting smoking. It is also possible that, as stress perception has been linked to withdrawal symptom intensity, the stress-buffering effects of exercise help smokers to cope with withdrawal (99). It should be noted that the findings of this review do not support that hypothesis.

All other outcomes were addressed by between one and three studies; for all outcomes reported by two or more studies, results were mixed, except in the case of depression, for which no study reported improvements. Overall, these findings reflect reviews and meta-analyses of all forms of exercise in nicotine dependence (23, 25, 100), namely that current study quality prohibits clear statements about effectiveness. Specifically, “Further studies are required to establish the optimum intensity of exercise intervention required […]” (23).

Only one study which may have involved anaerobic exercise in the treatment of alcohol dependence was identified. This study found no improvement with regard to excessive alcohol consumption in comparison to controls. A review of exercise interventions in SUDs noted that studies with alcohol dependent participants are more scarce than those assessing nicotine dependence (31), and in their summary of pre-clinical and clinical evidence for exercise in SUDs, Bardo and Compton note that findings in experimental studies appear to produce fewer promising results than those with nicotine or illicit drug dependence (101). This is in line with pre-clinical evidence suggesting that exercise does not appear to reliably decrease voluntary alcohol consumption. The authors suggest that the caloric content of alcohol may explain this phenomenon, as social factors are unlikely to be solely responsible given the findings of animal studies. Finally, Hallgren et al., in their meta-analysis of all forms of exercise in alcohol dependence, report that alcohol consumption is not reduced by exercise participation (30). Studies which compare alcohol dependent participants with individuals suffering from other SUDs are recommended, in order to explore whether the above findings are simply a result of limited, low-quality studies, or whether a genuinely different response to exercise among different SUDs exists.

Among the 13 studies which involved dependence on various illicit drugs, it is notable that depression and anxiety, addressed by six and five studies, respectively, were more commonly assessed than outcomes relating directly to consumption. Two studies reported abstinence, and three reported consumption. Results for these and all other outcomes assessed by two or more studies were mixed, with the exception of withdrawal symptoms, for which two studies reported reductions. In their meta-analysis, Wang et al. reported that exercise (all forms) appears to positively affect abstinence, withdrawal symptoms, and anxiety and depression. As with alcohol and nicotine, more studies which involve anaerobic activity are required to assess effectiveness, especially in comparison to aerobic exercise. However, it may be the case that individuals dependent on illicit drugs are generally in a poorer state of health than those dependent solely on nicotine (102), and consequently, anaerobic training may pose special challenges in this population.

It is important to note that the meta-analyses and reviews mentioned above include a number of the studies synthesized in this review, but do not evaluate them based on training intensity. This review suggests that anaerobic training may be somewhat more promising for nicotine dependence, and somewhat less promising for illicit drug dependence, although the likelihood of interaction and confounding between exercise forms means that, again, no conclusions can be drawn. While purely speculative, it is likely that nicotine dependent individuals are frequently in a generally better state of physical health than drug dependent individuals; consequently, nicotine dependent individuals may absorb and adapt to anaerobic training, and therefore benefit from it, while drug dependent individuals may not be able to adequately recover from, and hence adapt to, this form of training.

## Summary of Main Findings Regarding Exercise Categorization

Ten of the studies included in this review specifically state that the exercise form employed (not including comparison or control conditions) was aerobic, with six using the term “vigorous aerobic.” Only a single study determined the IAT of its participants; in all other cases, it is not possible to determine whether participants achieved or surpassed this threshold. In the literature on exercise in the treatment of SUDs, the term “aerobic” is currently employed to cover a broad range of exercise forms, in some instances without reference to a definition of the term itself. This lack of clarity means that it is difficult to determine what form of exercise has been employed in these studies, and consequently, whether different exercise types have different effects on relevant outcomes. This issue is not unique to the literature on SUDs (103), and it may be that concise definitions for various healthcare disciplines need to be developed, in order to improve the quality of both research and implementation; if practitioners do not know exactly what form of exercise they are prescribing, they cannot know what is or is not working.

Aside from a lack of clarity regarding the definition of aerobic or anaerobic exercise, the intensity of exercise carried out is also inconsistently reported. Thirteen studies in this review report the intensity at which participants exercised, though in some cases these are subjective self-reports, which are likely to be less accurate than objective assessment methods (104). Interestingly, only two of the nine studies which found no positive changes in outcome were among these twelve. Within these 12 studies, however, it is only possible to determine the amount of time spent at the reported intensity in one case. In the majority of cases, it is not possible to extricate any potential specific effects of particular intensities, as a certain amount of each exercise session is likely to have been spent in aerobic exercise. In the single study to compare timed acute bouts, there was a reduction in craving compared to non-exercisers and low-intensity exercisers only; no difference was found in comparison to exercise at up to 75% of maximum heart rate (84). While a determination of the IAT may be overly burdensome for clinical practice, prescription and determination of exercise intensity by heart rate zone would allow for a simple and reproducible, if somewhat less accurate, assessment.

Anaerobic exercise is frequently a consequence of participation in team sports or games, which require short bouts of high intensity (such as sprinting for a ball) followed by a recovery phase (e.g., as other players take over the ball). This type of exercise may be far more enjoyable for participants than cycling on a stationary bike (105). Only two studies in this review reported using team sports or games (78, 87); of these, intensity was assessed in just one (78). Fourteen studies involved indoor cycling or treadmill use. There is a paucity of studies which assess exercise intensity during team sports, and examine the effects on SUD relevant outcomes. Enjoyable activities which involve cooperation with others may have a positive effect on psychological variables and social integration, two areas in with individuals suffering from SUDs frequently experience deficits or difficulties.

It is also important to emphasize that anaerobic training may be less enjoyable to many individuals. As Peluso et al. note in their review of studies addressing exercise and mood, periods involving a focus on anaerobic training are frequently reported to result in more negative affect among elite and highly trained athletes (106). This is in line with the strong evidence indicating that intense exercise is generally associated with negative affect (107), although only one study in this review assessed mood, and reported reductions in negative affect (65). It would certainly be undesirable to expose patients to exercise which is (a) not enjoyable and (b) ineffective; in the absence of studies which clearly define exercise intensity, and demarcate the phases of training spent at these intensities, this may currently be the case in clinical practice.

## Limitations

This review must be considered in light of a number of limitations. First, as noted above, the heterogeneity of study designs, participants, and outcomes means that for a number of outcomes, little data is available. As noted above, only stud with alcohol, and one or two studies for different specific drugs, fulfilled our criteria. The treatment forms which participants were undergoing during the study periods were not assessed, and this is likely to have an impact on a number of the outcomes reported here. The effects of nicotine consumption in the alcohol and drug dependent populations was also not assessed. Information that would allow a more accurate risk of bias calculation could not be obtained for several articles.

## Conclusions

Currently, the evidence for anaerobic exercise in the treatment of SUDs is weak, although a tendency toward positive effects on abstinence in nicotine dependent individuals was observable. This is in line with reviews which address exercise in general in this domain; while individual studies sometimes show positive effects, the quality of the evidence prohibits clear statements of effectiveness. Furthermore, few studies which involve more vigorous forms of exercise define and report the intensities achieved by participants. In most cases, this means that the effects of different intensities cannot be determined, and therefore cannot be evaluated or systematically implemented. In order to improve the quality of evidence for exercise in SUD treatment, clearly defined and objectively assessed evaluations of anaerobic and anaerobic exercise are necessary.

## Author Contributions

FC conceived the research question, carried out the review, and prepared the initial draft of the manuscript. MG mediated the review and synthesis process, and revised the manuscript. UP revised the manuscript. SL carried out the review and revised the manuscript.

### Conflict of Interest Statement

The authors declare that the research was conducted in the absence of any commercial or financial relationships that could be construed as a potential conflict of interest.
